# Targeting chemokines/chemokine receptors: a promising strategy for enhancing the immunotherapy of pancreatic ductal adenocarcinoma

**DOI:** 10.1038/s41392-020-00267-8

**Published:** 2020-08-11

**Authors:** Ruining Gong, He Ren

**Affiliations:** Department of Gastroenterology, Center of Tumor Immunology and Cytotherapy, Medical Research Center of The Affiliated Hospital of Qingdao University, 266003 Qingdao, China

**Keywords:** Gastrointestinal cancer, Tumour immunology

**Fig. 1:**
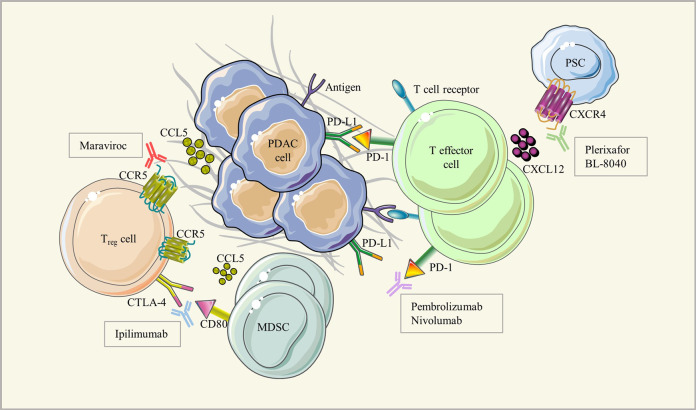
The interaction between immune and cancer cells and their targeting inhibitors to treat pancreatic ductal adenocarcinoma (PDAC). Pancreatic cancer cells secrete cytokines and chemokines to recruit stromal cells including myeloid-derived suppressor cells (MDSCs), regulatory T cells (Tregs) and pancreatic stellate cells (PSCs). Immune checkpoint inhibitors (ICI) including anti-PD-1/PD-L1 and anti-CTLA-4. Targeting chemokines/chemokine receptors like the CCL5/CCR5 and CXCL12/CXCR4 axis facilitates the immunotherapy of pancreatic ductal adenocarcinoma

**In recent study published on Nature Medicine, Bockorny et al.**^1^ **performed a single-arm phase IIa trial (COMBAT study, NCT02826486) to evaluate safety, efficacy, immunobiological changes, and potential biomarkers for the CXCR4 inhibitor BL-8040, combined with a PD-1 antagonist (pembrolizumab) as a second-line or third-line treatment for patients with metastatic PDAC. This evidence translates the theory of reprogramming tumor immunosuppressive microenvironment into clinical practice and supports that targeting chemokines/chemokine receptors facilitates the immunotherapy of pancreatic ductal adenocarcinoma (Fig.** 1**).**

Pancreatic ductal adenocarcinoma (PDAC) is one of the most lethal tumors with resistance to traditional treatments. Immune checkpoint inhibitors (ICI) have opened a new avenue in the treatment of multiple cancers; however, the mono-therapeutic effects of anti-PD-1/PD-L1 or anti-CTLA-4 are not satisfactory on PDAC. The prerequisite for an effective ICI therapy is high levels of activated tumor-infiltrating lymphocytes (TIL) in the tumor tissues (also called “hot” tumors). However, most PDACs are characterized by low levels of activated TIL around tumor tissues (also called “cold” tumors) due to the desmoplastic stroma and multiple immunosuppressive cells, such as regulatory T cells, M2 macrophages, and myeloid-derived suppressive cells.^2^ Therefore, strategies to convert the microenvironment from “cold” to “hot” by enhancing TIL levels and activities have been used in ICI-based combination trials.

CXC chemokine receptor 4 (CXCR4) belongs to the superfamily G-protein coupled receptors that is highly expressed in a variety of human cancers and is significantly correlated with poor prognosis. CXCL12 binds to CXCR4 to promote proliferation, migration, and angiogenesis of PDAC. Importantly, CXCL12/CXCR4 signaling reduces the TIL levels in the microenvironment of PDAC and mediates an immune escape. Pre-clinical and clinical studies have indicated that the CXCR4 blockade enhances the infiltration of TIL and reduces the immunosuppressive cells in the tumor microenvironment, thus converting the tumor from “cold” to “hot.” BL-8040 is a high-affinity peptide with a long receptor occupancy of CXCR4. It has been tested in multiple pre-clinical models to demonstrate effective mobilization of bone-marrow-derived lymphocytes and selective reduction of regulatory T cells.

The clinical trial included two cohorts. Among the 16 patients receiving the combination treatments as a second-line treatment, the median overall survival was superior to that of the previous US Food and Drug Administration approved NAPOLI-1 regimen (liposomal irinotecan, fluorouracil, and leucovorin) (7.5 months vs. 6.1 months).^3^ In cohort 2, 22 metastatic patients who have progressed after gemcitabine treatment received triple combination strategies, including BL-8040, Pembrolizumab, and the NAPOLI-1 regimen. Notably, the disease control rate reached an encouraging level of 77%, and the average effective duration was 7.8 months. This presented an encouraging progress for the treatment of PDAC.

Microsatellite instability-high (MSI-H) has been identified as a biomarker to predict responses to the PD-1 blockade. MSI is related to deficiencies in DNA mismatch repair genes, which results in the generation of mutation-associated neoantigens.^4^ However, patients with MSI-H are rare and close to 1–2%, and for these patients, pembrolizumab has been approved by the Food and Drug Administration. Interestingly, none of the 22 patients in the second cohort of this clinical trial were determined to have MSI-H. This may suggest that inhibitors of chemokine receptors may act as sensitizers to improve the effects of ICI, independent of MSI-H.

In the same study, the level of carbohydrate 19-9 antigen elevated early and significantly decreased at a later stage. This suggests that it may not be appropriate as an early indicator of efficacy. It should be noted that BL-8040 plus pembrolizumab reduces circulating PD-1^+^CD4^+^ T cells among patients with disease control. This indicates that circulating PD-1^+^CD4^+^ T cells can be a candidate non-invasive biomarker for monitoring responses to immuno-therapeutic strategies. The repeated biopsies to verify immunobiological changes are consistent with the pre-clinical data from the mice models of PDAC, further confirming that the theory of reprogramming tumor microenvironment is promising for the ICI-based combination strategies in practice.

Importantly, multiple reagents for the PDAC treatment were well tolerated, and only two patients discontinued due to the side effects. The incidence of grade 3–4 adverse events was relatively low and indicated that targeting the chemokines/chemokine receptors might be a good strategy for enhancing the ICI-based therapy without an obvious increase in side effects. It may also reduce intolerance for elderly patients, who comprise the majority of PDAC cases. However, the feasibility of combinations with BL-8040 and PD-1 remains to be further evaluated with subsequent analysis from randomized double-blinded controlled clinical trials.

Recently, we found that combinations with CCL5/CCR5 antagonist and the anti-PD-L1 antibody inhibited tumor growth and improved overall survival in mice models of PDAC.^5^ Here, we first determined that cancer-FOXP3 mediated immune escape by recruiting regulatory T cells via upregulation of CCL5. We provided the rationale that cancer-FOXP3 could identify PDAC patients suited for combination of ICI and the chemokines/chemokine receptors inhibitor, in order to improve response. Interestingly, CCR5 and CXCR4 serve as the co-receptors for HIV-1 entry into T cells, and they have been used as popular targets for the development of new drugs. BL-8040, the peptide-based motixafortide, exhibits encouraging results in combination treatment. Meanwhile, the CCR5 antagonists, such as the small molecular reagents like maraviroc and the humanized monoclonal anti-CCR5 antibody leronlimab, have achieved the primary endpoints in phase 3 clinical studies on HIV, and therefore could be considered for therapeutic repurposing in PDAC.

In the future, additional studies on incorporating the CCL5/CCR5 and CXCL12/CXCR4 pathway blockade for the treatment of PDAC may overcome the compensation mechanism of single-pathway chemokines/chemokine receptors and encourage the innovation of therapeutic strategies for the lethal disease.
